# Rectal diaphragm in a patient with imperforate anus and rectoprostatic fistula

**DOI:** 10.4103/0971-9261.54814

**Published:** 2009

**Authors:** Ashokanand Thakur, N. P. Dhende, S. B. Mane, Himanshu Acharya

**Affiliations:** Department of Pediatric Surgery, Sir J J Group of Hospital, Mumbai - 400 008, India

**Keywords:** Ano-rectal malformation, imperforate anus, rectal diaphragm

## Abstract

The association of rectal diaphragm in an imperforate anus has not been reported until now. A 1-year-old male presented with right transverse colostomy for high anorectal malformation. The patient had imperforate anus and a recto-prostatic fistula with rectal diaphragm. We managed the case by an ano-rectal pull through with excision of the diaphragm.

## INTRODUCTION

A rectal diaphragm with ano-rectal agenesis has not been reported until now. There are reports of a rectal diaphragm in the cases of rectal atresia where there is a normal anal canal. We are reporting a patient with a rectal diaphragm with imperforate anus and rectoprostatic fistula. The patient was suspected and managed initially as a case of high anorectal malformation.

## CASE REPORT

A 1-year-old male presented to us with an absent anal opening and transverse colostomy, which was performed on day 2 of life at another hospital. Upon clinical examination, he had a flat bottom, two sacral pieces, and absent anocutaneous reflexes, which are consistent with high anorectal malformation.

**Figure 1:**
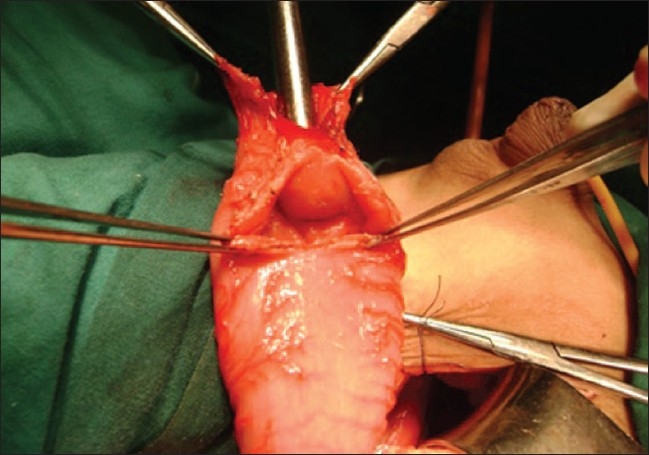
Rectal diaphragm

An ultrasound of the abdomen showed single kidney on the left side. A micturating cystourethrogram showed grade IV reflux with anterior diverticulum of the urethra. A distal cologram was showing a distended colon without any fistula [Figure 1]. A renal scan confirmed the absence of the right kidney with a normal functioning left kidney.

**Figure 2:**
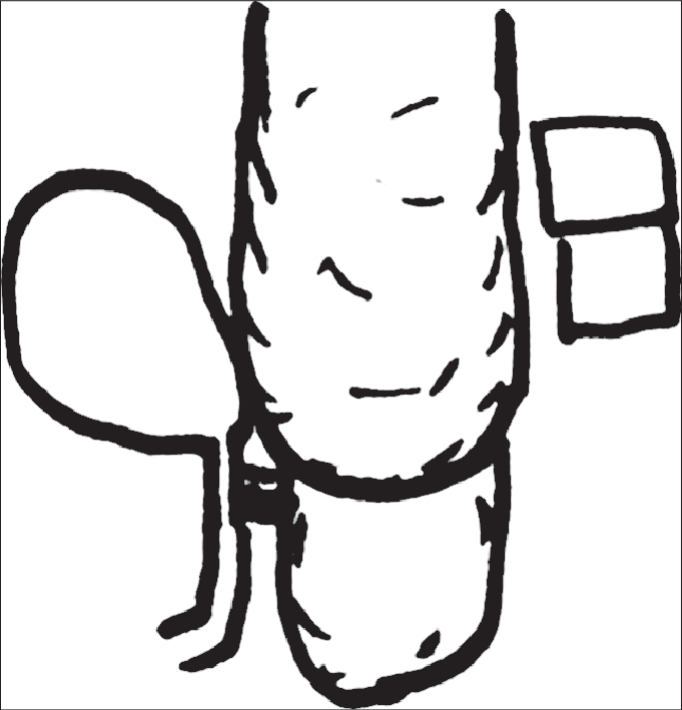
Schematic diagram of rectal diaphragm in imperforate anus with delineating fistula and only two sacral pieces

Because of the single kidney with reflux, the patient initially underwent Cohen's transtrigonal ureteric reimplantation along with an excision of the dorsal diverticulum of the anterior urethra. After 2 months, the patient was scheduled for a posterior saggital anorectoplasty (PSARP). Intraoperatively, after opening the sphincter muscle, a complex rectal pouch was identified. The rectal pouch was opened at the midline at the distal most summit as done in routine posterior saggital anorectoplasty. This revealed a blind pouch with a recto-prostatic fistulous opening, which was an unusual finding for us. We tried to mobilise the rectum from below in the sub mucosal plane as further operation was not possible from the posterior route. The decision was made to mobilize the rectum through the abdominal route. The abdomen was opened by a left hockey stick incision and the rectum was mobilized. The rectum was dilated proximally; on opening of rectum, the diaphragm was found about 2 cm proximal to the distal end [Figure 2]. The terminal end of the rectum along with diaphragm was excised and a closure of the fistula was performed. The dilated rectum was tapered and an anoplasty done.

A histopathological examination of the excised specimen was consistent with the diagnosis of diaphragm. The patient is on regular follow-up of 8 months with no complaints. He underwent a colostomy closure 2 months ago with no complaints in the post-operative period.

## DISCUSSION

The rectal diaphragm in anorectal agenesis with rectoprostatic fistula is a rare association. A search of the online database did not show any such reference. There are isolated case reports of a rectal diaphragm with or without fenestration with a normal anal opening. We propose that this is a rare and probably the first case report with such an association.

Embryologically the rectal diaphragm is included in a type of rectal atresia.[1] The embryology states that rectal atresia is due to a vascular accident occurring antenatally. The histology of the diaphragm shows a thin sheet of fibrous tissue in which there is some smooth muscle.[2]

Ano-rectal malformation is secondary to failure in differentiation of cloaca antenatally. Thus, the embryological correlation between ano-rectal malformation and rectal diaphragm is difficult to prove.

Clinical presentation of this association is the same as that for any ano-rectal malformation in the neonatal period. Thus, it is difficult to diagnose this association in the neonatal period. The anomaly is difficult to diagnose on a distal cologram as it mimics the ano-rectal malformation without fistula. The rectal diaphragm with ano-rectal agenesis can be diagnosed intraoperatively during posterior saggital ano rectoplasty.[3]

**Figure 3:**
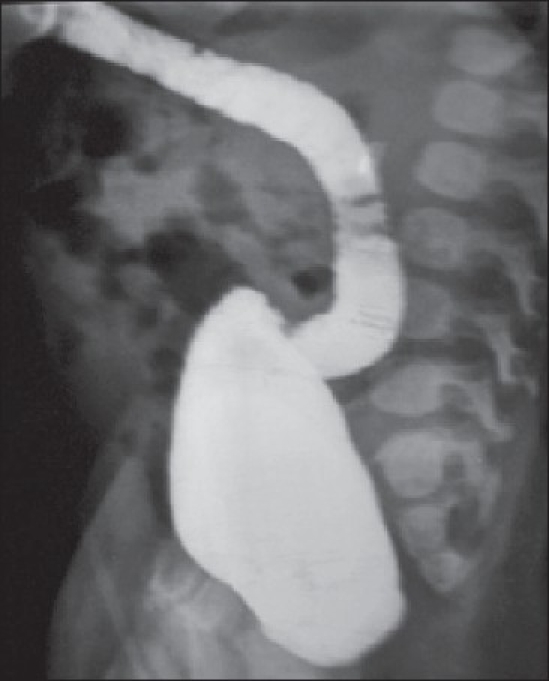
Distal cologram showing blind ending rectum with faecoloma

During PSARP, when the rectum is opened to locate the fistula, the diaphragm can be seen. In our case, the diaphragm was located proximal to the site of the fistula. In case the rectum is opened at its distal most limits, one finds the blind pouch and no proximal continuity of the lumen [Figure 3]. In such cases, a surgeon may become confused with a condition akin to the accidental opening of the urinary bladder. However, if the bladder is opened, the urinary catheter should be seen. If the urinary catheter is not seen, then rectal pouch incision should be extended proximally to find the proximal lumen of the rectal pouch. However, in our case, we closed the rectal pouch incision and abdominal PSARP with tackling of the rectoprostatic fistula. In our case, the rectal diaphragm was excised along with the distal rectum and tapering of the rectal pouch and an anoplasty was done. Postoperatively, the patient recovered well.
